# Introduction of Mouse Embryonic Fibroblasts into Early Embryos Causes Reprogramming and (Con)fusion

**DOI:** 10.3390/cells10040772

**Published:** 2021-03-31

**Authors:** Pierre Savatier

**Affiliations:** Stem Cell and Brain Research Institute U1208, Inserm, Univ Lyon, Université Lyon 1, 69500 Bron, France; pierre.savatier@inserm.fr

The reprogramming of somatic cell nuclei to achieve pluripotency is one of the most important biological discoveries of the last few decades. This milestone has been achieved by somatic cell nuclear transfer (SCNT) into enucleated oocytes to generate nuclear transfer embryonic stem (ntES) cells and cloned animals [1,2] as well as cell–cell fusion between somatic cells and embryo-derived pluripotent stem cells lines to generate hybrid cells [3]. Ultimately, by direct reprogramming with transcription factors, this paradigm has culminated in the generation of induced pluripotent stem (iPS) cells [4]. In mice, ntES, hybrid and iPS cells can form systemic chimeras when introduced into preimplantation embryos, confirming their intrinsic pluripotentiality [2,5,6].

In the last issue of *Cells*, Żyżyńska-Galeńska et al. [7] reported that mouse morulae provide a favorable niche to reprogram mouse embryonic fibroblasts (MEFs) to express pluripotency markers and participate in adult chimeras. The experimental paradigm is based on the introduction of 3–4 red fluorescence labelled MEFs in the centre of the 8–16 cell stage mouse embryos, followed by in vitro culture for two days or transfer to surrogate females. Results suggest that two different reprogramming mechanisms occur in the preimplantation embryos. First, niche-induced reprogramming results in MEF-derived cells expressing early lineage markers, including the early endodermal marker Pdgfra and Gata4, the early trophectoderm marker Cdx2, and the pluripotency markers Nanog as well as Oct4, the latter evidenced by *Oct4* promoter reactivation in MEF nuclei. The authors provide data indicating that most MEF progeny observed in trophectoderm, primitive endoderm, and inner cell mass (ICM) express Cdx2, Pdgfra/Gata4, and Nanog, respectively. These findings strongly suggest that MEF tend to adopt the fate of the lineage they have colonized. Note that 13% of the MEF progeny expressed both Nanog and Gata4, two mutually exclusive markers of epiblast and primitive endoderm, respectively. These data suggest that some of the MEF derivatives were mis-specified. The authors did not discuss the mechanism by which MEFs can be reprogrammed to express markers of the three blastocyst lineages. One may be tempted to speculate that blastomeres can alter a cell phenotype through cell–cell interactions, a mechanism that remains to be proven in this context. A more likely explanation is that cell microinjection destroys one or more blastomeres in the host embryos, resulting in the release of cytoplasmic determinants in the embryonic environment and triggering reprogramming in a process comparable with SCNT. This explanation is supported by the authors’ observation that reactivation of the lineage markers is only observed when MEFs are introduced inside the morula, a procedure that inevitably leads to blastomere damage, whereas reactivation of these markers is not observed when MEFs are deposited at the periphery of the embryo under the zona pellucida. Regardless of the mechanism, MEFs are presumably reprogrammed to pluripotency within 24 hours of their introduction into the morula environment. Many then seem to adopt trophectoderm-, endoderm- or epiblast-like fates depending on their spatial allocation. The question is whether these reprogrammed cells take part in the development of the fetus. The authors reported both the persistence of donor cell DNA in the E13.5 fetuses and the detection of copious red fluorescence-emitting cells in the brain and heart of the adult mice. To ascertain the pluripotency of the reprogrammed MEFs, studies must still address whether these cells express markers of ectoderm, mesoderm, and definitive endoderm.

The authors reported that the introduction of MEFs into the 8–16-cell stage mouse embryos also resulted in cell fusion, as evidenced by the hybrid cells expressing both red (MEFs) and green (host) fluorescence. In the blastocysts, hybrid cells accounted for 42% of MEF-derived cells, with niche-induced reprogramming described above accounting for the remaining 58%. Unfortunately, these hybrid donor/host cells have not been systematically characterized for the expression of lineage markers, leaving their identity uncertain. Particularly, the results are unclear regarding whether these hybrid donor/host cells express pluripotency or lineage markers that correspond to their spatial allocation, unlike the clearer results shown for the niche-induced cells. To assess the hybrid cells’ contribution to fetal development, the authors examined the tetraploidy that results from cell fusion. Hybrid tetraploid cells were detected in virtually all fetal and adult tissue samples, with rates ranging from 0.7% to 25% of all MEF-originating cells. The results are unclear regarding whether these tetraploid cells resulted from the persistence of the MEF-derived hybrid cells identified in the blastocysts as previously shown [8] or from de novo fusions taking place during organogenesis.

The results reported by Żyżyńska-Galeńska et al. strongly suggest that mouse embryo blastomeres can reprogram the nucleus of an embryonic fibroblast, resulting in immature cells with an inactivated senescence programme. Although some cells re-expressed Nanog and Oct4, a more indepth analysis is required to demonstrate their true pluripotency. Notably, 71% of the foetuses that developed from embryos with confirmed MEF contribution were growth-retarded, abnormal, or resorbed. This statistic strongly suggests that even a low contribution of MEF-derived cells harms development. Nevertheless, the authors provide new information about somatic cell reprogramming, highlighting previously unknown phenomena that occur inside the embryo after introducing somatic cells.

Arguably, the findings of this paper shed new light on interspecies chimeras, systemic chimeras produced by introducing the embryo-derived or induced pluripotent stem cells (PSC) of one species, usually human or rhesus monkey, into the preimplantation embryos of a different species, typically mouse, rabbit or pig [9,10,11,12]. Chimeric competence is a functional criterion for defining the naive state of pluripotency. Interspecies chimerism is often used to assess pluripotent stem cell lines for this quality. The results are highly variable and sometimes not reproducible. Additionally, ongoing confusion persists regarding whether human PSCs are truly competent to form interspecies chimeras. The report by Żyżyńska-Galeńska et al. [7] adds further complexity to an already complex experimental paradigm. Chimeric-incompetent PSCs could become chimeric-competent through either blastomere damage or cell fusion; both incidents would occur after introduction into early embryos and would hide the true nature of the original cells. Additionally, cell fusions between donor and host cells could occur throughout organogenesis, producing hybrid cells with confusing phenotypes. For example, a seemingly differentiated donor cell could actually be a tetraploid cell composed of a host nucleus expressing lineage-specific markers and a mis-differentiated or undifferentiated donor nucleus. Thus, the report by Żyżyńska-Galeńska et al. signifies the need to closely examine the characterization of chimeras before drawing conclusions about naive pluripotency and harmonious chimerism in nonrodent species.
